# AMPK Activation Alleviates Myocardial Ischemia-Reperfusion Injury by Regulating Drp1-Mediated Mitochondrial Dynamics

**DOI:** 10.3389/fphar.2022.862204

**Published:** 2022-07-04

**Authors:** Jingxia Du, Hongchao Li, Jingjing Song, Tingting Wang, Yibo Dong, An Zhan, Yan Li, Gaofeng Liang

**Affiliations:** ^1^ Pharmacy Department, School of Basic Medical Sciences, Henan University of Science and Technology, Luoyang, China; ^2^ Pathology Department, School of Basic Medical Sciences, Henan University of Science and Technology, Luoyang, China

**Keywords:** AMPK, DRP1, mitochondrial dynamics, myocardial ischemia/reperfusion injury, ROS, inflammatory factors

## Abstract

Mitochondrial dysfunction is a salient feature of myocardial ischemia/reperfusion injury (MIRI), while the potential mechanism of mitochondrial dynamics disorder remains unclear. This study sought to explore whether activation of Adenosine monophosphate-activated protein kinase (AMPK) could alleviate MIRI by regulating GTPase dynamin-related protein 1 (Drp1)-mediated mitochondrial dynamics. Isolated mouse hearts in a Langendorff perfusion system were subjected to ischemia/reperfusion (I/R) treatment, and H9C2 cells were subjected to hypoxia /reoxygenation (H/R) treatment *in vitro*. The results showed that AICAR, the AMPK activator, could significantly improve the function of left ventricular, decrease arrhythmia incidence and myocardial infarction area of isolated hearts. Meanwhile, AICAR increased superoxide dismutase (SOD) activity and decreased malondialdehyde (MDA) content in myocardial homogenate. Mechanistically, AICAR inhibited the phosphorylation of Drp1 at Ser 616 while enhanced phosphorylation of Drp1 at Ser 637. In addition, AICAR reduced the expression of inflammatory cytokines including *TNF-ɑ, IL-6,* and *IL-1β*, as well as mitochondrial fission genes *Mff* and *Fis1*, while improved the expression of mitochondrial fusion genes *Mfn1* and *Mfn2*. Similar results were also observed in H9C2 cells. AICAR improved mitochondrial membrane potential (MMP), reduced reactive oxygen species (ROS) production, and inhibited mitochondrial damage. To further prove if Drp1 regulated mitochondrial dynamics mediated AMPK protection effect, the mitochondrial fission inhibitor Mdivi-1 was utilized. We found that Mdivi-1 significantly improved MMP, inhibited ROS production, reduced the expression of *TNF-a, IL-6, IL-1β, Fis1*, and *Mff*, and improved the expression of *Mfn1* and *Mfn2*. However, the protection effect of Mdivi-1 was not reversed by AMPK inhibitor Compound C. In conclusion, this study confirmed that activation of AMPK exerted the protective effects on MIRI, which were largely dependent on the inhibition of Drp1-mediated mitochondrial fission.

## Introduction

Acute myocardial infarction (AMI) is the global leading cause of death in cardiovascular diseases (Tibaut et al., 2017). Restore blood supply in time to ischemic myocardium is the most effective treatment for AMI. Reperfusion is a viable therapeutic strategy for ischemic heart disease with high morbidity and mortality (Ren et al., 2021). However, reperfusion may also exacerbate myocardial damage and produce a second blow to the myocardium, such as excessively generating reactive oxygen and nitrogen species (Daiber et al., 2021), increasing release of inflammatory mediators and recruitment of inflammatory cells (Toldo et al., 2018), mitochondrial Ca^2+^ overload, and the opening of the mitochondrial permeability transition pore (MPTP) (Ong et al., 2015; Basalay et al., 2020). Cardiomyocytes are rich in mitochondria, and mitochondrial dysfunction, which induces cardiomyocytes apoptosis or necrosis, eventually leads to heart injury and dysfunction (Zhang et al., 2021a). Although AMI patients may receive timely reperfusion therapy, reperfusion after revascularization of an AMI can contribute up to 50% of the resulting infarct damage (Levent et al., 2020). Therefore, exploring the molecular mechanism and new strategies for MIRI intervention are greatly needed.

Mitochondrial dysfunction is closely related to metabolic disorders, ischemic heart disease, and many other diseases (Xue et al., 2020). During MIRI, mitochondria ATP production and mitochondrial membrane potential (MMP) are decreased, while reactive oxygen species (ROS) are excessively produced, which collectively lead to myocardial damage (Hou et al., 2018). Therefore, preventing the detrimental effects of mitochondria is an important therapeutic strategy for MIRI.

AMPK, a highly conserved serine/threonine-protein kinase and also a key regulator of cellular homeostatic balance, is activated during metabolic cellular stress such as decreased oxygen or glucose supply, or increased AMP/ATP ratio (Hardie et al., 2012). Intrinsic activation of AMPK is extremely important to hold back excess mitochondrial ROS production and consequent JNK signaling activation during reperfusion (Zaha et al., 2016). Recently, sappanone A (SA) postconditioning was demonstrated to ameliorate MIRI by regulating mitochondrial quality control *via* activating AMPK (Shi et al., 2021). Calenduloside E (CE) is effective in mitigating MIRI by modulating AMPK-mediated OPA1-related mitochondrial fusion (Wang et al., 2020). These studies strongly suggest that the activation of AMPK plays a crucial role in the prevention of MIRI by regulating the mitochondrial function.

Mitochondria are highly dynamic organelles, constantly undergoing dynamic changes controlled by mitochondrial fusion and fission (Hsu et al., 2021). Disruption of dynamic balance always leads to heterogeneity and dysfunction of mitochondria (Zhang et al., 2021b). Mitochondrial dynamics are regulated by fusion and fission genes. Mitochondrial fusion is regulated by a family of GTPases including *Mfn1* and *Mfn2* (Hall et al., 2016), while mitochondrial fission is largely regulated by the *Drp1* and the Drp1 targeting molecule fission 1 (*Fis1*) (Vongsfak et al., 2021). Drp1, located in the cytosol, is essential for mitochondrial fission (Bradshaw et al., 2016). During mitochondrial fission, cytosolic Drp1 is recruited to mitochondria, binds to adaptors on mitochondrial outer membranes, and assembles into oligomers to constrict and sever mitochondrial membranes (van der Bliek et al., 2013; Yu et al., 2021). The most characteristic mechanism of post-translational modification in Drp-1 involves phosphorylation at two key sites: Drp1 (Ser616) and Drp1 (Ser637) (Chang and Blackstone, 2010). Excessive mitochondrial fission results in the collapse of membrane potential, the elevation of ROS production, and consequently cellular injury or death (Xu et al., 2017).

Even though AMPK may play a crucial role in the prevention of MIRI, it is largely unknown whether AMPK activation alleviates MIRI by regulating Drp1-mediated mitochondrial dynamics. Herein, a mouse model of MIRI *in vitro* and a H9C2 cell model of H/R were established to investigate whether AMPK activation alleviates MIRI by regulating Drp1-mediated mitochondrial dynamics.

## Materials and Methods

### Animals and H9C2 Cell Culture

The C57BL/6J mice (male, 20–25 g) were obtained from the Laboratory Animal Center of Henan University of Science and Technology (Luoyang, China, License number: SCXK 2020-0008). All the animal experiments were approved by the Animal Care and Ethics Committee of Henan University of Science and Technology (Luoyang, China) and performed in compliance with the guidelines for the Principles of Laboratory Animal Care and Use of Laboratory Animals published by NIH (NIH Publication, 8th Edition, 2011).

H9C2 cells were purchased from ATCC, and were cultured in a mixture of 10% fetal bovine serum (FBS) and double antibiotics (penicillin and streptomycin, 100 μg/ml) in Dulbecco’s modified eagle medium (DMEM).

### Langendorff Perfused Heart I/R Model

Mice were intraperitoneally injected with ethyl urethane (1 g/kg) for anesthesia, and heparin (500 IU/kg) for heparinization. The hearts were taken rapidly from the thoracic cavity and fixed on the Langendorff apparatus. Isolated hearts were either continuously perfused in the control group, or balanced for 15 min, stopped perfusion for 30 min, and reperfused for 30 min to replicate the I/R experimental model. The detailed process of the MIRI model was shown in the supplementary file, Supplementary Figure S1. Retrograde coronary perfusion was maintained with Tyrode solution (g/L, NaCl 7.895, KCl 0.403, MgCl_2_•6H_2_O 0.203, CaCl_2_ 0.200, NaH_2_PO_4_•2H_2_O 0.052, Hepes 2.383, and glucose 1.982, PH 7.35–7.45) at constant pressure in the temperature controlled glass chamber (37.5°C). A latex balloon connected to the pressure transducer was inserted into the left ventricle. The BL-420 S biological and functional experimental system was used for monitoring heart rate, left ventricular peak pressure (LVSP), and electrocardiogram (ECG) continuously.

### Hypoxia/Reoxygenation Model in H9C2 Cells

H9C2 cells with hypoxia /reoxygenation (H/R) to mimic the I/R model *in vitro*. The hypoxic condition was achieved *via* a hypoxic incubator (95% N_2_ and 5% CO_2_). The conventional culture medium was replaced with serum-free medium (100% DMEM) and incubated for 12 h in an incubator at 37°C with 95% N_2_ and 5% CO_2_. Thereafter, the serum-free medium (100% DMEM) was then replaced with the conventional culture medium (90% DMEM + 10% FBS), and the cells were incubated for 12 h reoxygenation at 37°C in 95% air and 5% CO_2_. The cells were pre-incubated with AICAR (1 mM), Compound C (25 μM) or Mdivi-1 (50 μM) for 1 h if necessary.

### TTC Staining for Myocardial Infarction Size Measurement

The hearts were immediately taken down from the Langendorff perfusion device at the end of reperfusion, and cut into pieces about 2 mm thick, and then placed in 1% triphenyltetrazolium chloride (TTC, lot number: abs47011070, Absin) for 20 min. Then these pieces were photographed, and the area of the non-infarct area (red) and infarct area (white) was calculated by Image-Pro Image analysis software. The percentage of infarct area to the total myocardial area was calculated according to the following formula:

$$\text{Myocardial infarct area }\left(\text{\%}\right)=\frac{\text{myocardial infarct area}}{\text{total myocardial area}}\times 100\text{\%}$$

### Creatine Kinase and Lactate Dehydrogenase Activity Measurement

Myocardial injury was reflected by the activity of CK and LDH in the coronary effluent or the supernatant of H9C2 cell culture by using CK and LDH assay kit respectively according to the manufacturer’s instructions (Nanjing Jiancheng Biotechnology, China).

### Superoxide Dismutase Activity and Malondialdehyde Content Measurement

Tissue homogenate (10%, weight/volume) was made from part of left ventricular and centrifuged at 4°C, 3,000 rpm for 15 min, and the supernatant was obtained to detect the SOD activity and MDA content according to the manufacturer’s instructions (Nanjing Jiancheng Bioengineering Institute, China).

### Intracellular ROS Assay

For intracellular ROS detection, H9C2 cells were treated with H/R, and then incubated with ROS specific fluorescent probe dye DCFH-DA (Beyotime Institute of Biotechnology, Shanghai, China) for 20 min at 37°C. Flow cytometry was performed to analyze the levels of mitochondrial ROS.

### Mitochondrial Membrane Potential Analysis

A mitochondrial membrane potential assay kit with JC-1 (Beyotime Institute of Biotechnology, Shanghai, China) was used to detect changes in the mitochondrial membrane potential by confocal laser scanning microscope (CLSM) and flow cytometry, which were performed according to the manufacturer’s protocol.

### Mitochondrial Morphological Detection by Transmission Electron Microscopy

After finishing the H/R process, the cells were collected and immediately fixation in 2.5% glutaraldehyde for 2 h, and then soaked in 0.1 M phosphoric acid buffer for 30 min, fixed in 1% osmium tetroxide solution at 4°C for 2 h. After that, cells were dehydrated using a graded ethanol immersion series and then embedded in resin. The H9C2 cells were cut into 50–70 nm sections using an ultramicrotome (UC7, Leica), and stained with uranium acetate and lead citrate, and finally observed using an electron microscope (H-7650, HITACHI).

### Western Blot Analysis

The tissue or cell total proteins were extracted with lysis buffer (RIPA) containing protein phosphatase inhibitor (Beijing Solar Science & Technology Co., Ltd.). As reported, the quantity of protein was measured by the BCA method (Sangon Bioengineering, Shanghai). Denatured protein samples (30 μg) were subjected to SDS-PAGE. After electrophoresis, protein was transferred to PVDF membranes, which were then blocked for 1 h by 5% nonfat dry milk in TBS-T (mM, Tris-base 24.8, NaCl 136.8, KCl 2.7, 0.1% Tween-20, pH 7.4) and subsequently incubated with primary antibody in 5% nonfat dry milk at 4°C overnight. After washing thrice with TBS-T, the membranes were incubated with anti-rabbit or anti-mouse horseradish peroxidase-conjugated secondary antibodies (1:5000 dilutions) for 1 h. The signal was detected by chemiluminescence using the ECL detection system (Amersham) (Du et al., 2020). The same procedure was repeated for AMPK (1:1000), p-AMPK (1:1000), p-Drp1 (Ser 616) (1:1000), p-Drp1 (Ser 637), (1:1000), Drp1 (1:1000), and GAPDH (1:5000) at 4°C overnight. GAPDH was used as a loading control for total protein. Quantification of bands was performed using ImageJ Software (NIH).

### Quantitative Real-Time Polymerase Chain Reaction

Total RNA was extracted from the tissue or cells using TRIzol reagent (TaKaRa Bio, Dalian, China) and reverse-transcribed using a high-capacity complementary DNA reverse transcription kit (CWBio, Beijing, China). qPCR was performed on the 7,500 Sequence Detection System (Applied Biosystems, Foster City, CA, United States ) by using SYBR™ Green PCR Master Mix kit (CWBio, Beijing, China) according to the manufacturer’s protocol. The reaction system was as follows: 10 min at 95°C and then 15 s at 95°C and 1 min at 60°C for 40 cycles. The expression of inflammatory factor including tumor necrosis factor-α (*TNF-α*), Interleukin-6 (*IL-6*), Interleukin-1β (*IL-1β*), and mitochondrial dynamics regulators including *Fis1*, *Mff*, *Mfn1*, and *Mfn2* were quantified. The primers used in the present study were listed in supplementary file Supplementary Table S1. The 2^−ΔΔCt^ method was used to quantify the genes, and *β-actin* gene was used as the internal control.

### Statistical Analysis

Data were expressed as means ± SEM. All experimental data were analyzed by using a one-way analysis of variance (ANOVA) followed by a Tukey multiple comparison test. A level of *p* < 0.05 was considered to be a statistically significant difference.

## Results

### Activation of AMPK Alleviated MIRI in Isolated Mouse Hearts

**FIGURE 1:**
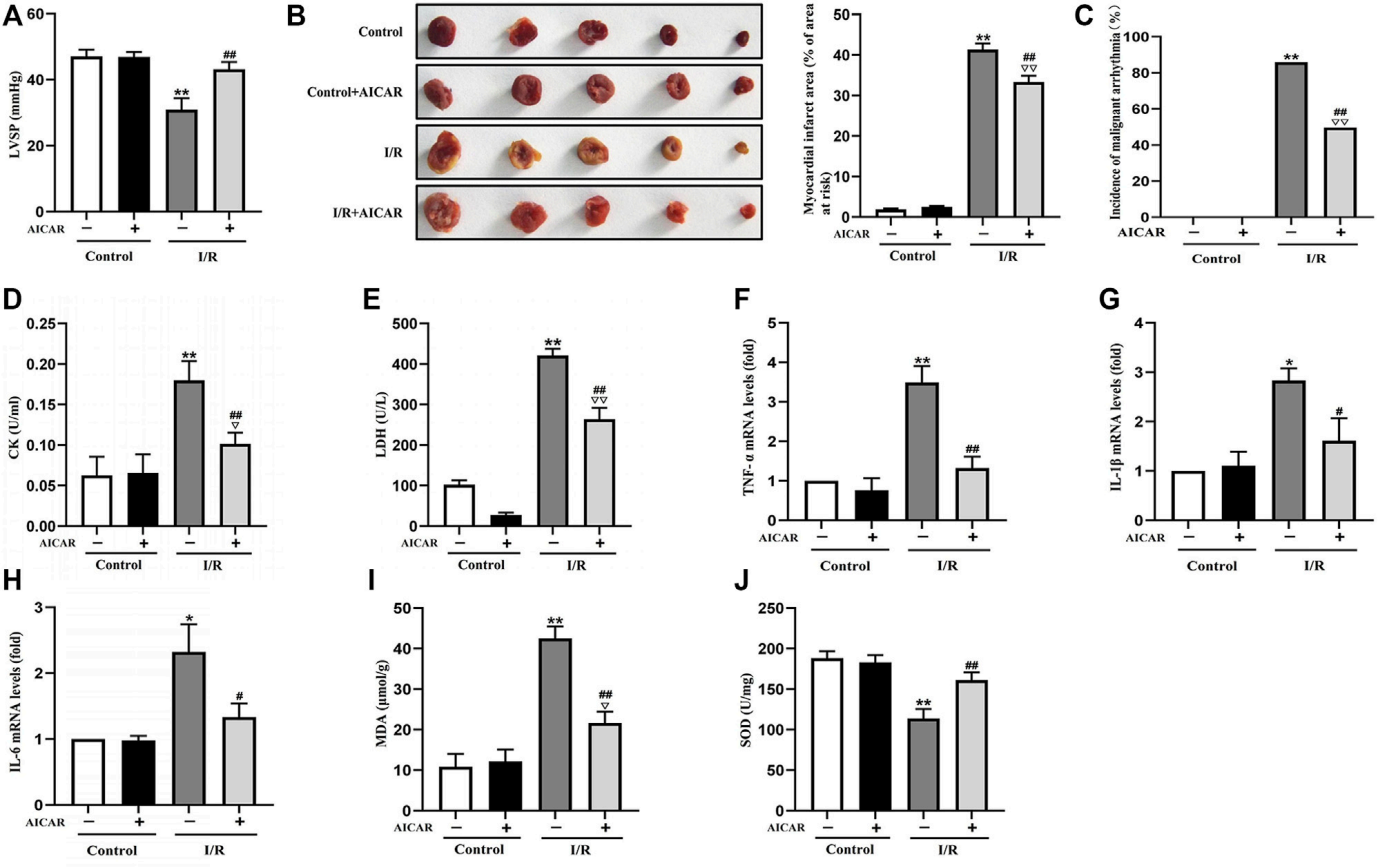
Activation of AMPK alleviated MIRI in isolated mouse hearts. **(A)** AICAR improved the LVSP. **(B)** Representative images of tissue sections stained with TTC to detect the myocardial infarct size. **(C)** AICAR decreased the incidence of malignant arrhythmia. **(D,E)** AICAR decreased the levels of CK and LDH in coronary effluent fluid. **(F–H)** AICAR decreased the mRNA expression of *TNF-α, IL-1β* and *IL-6* in myocardium tissue. **(I,J)** Measurement of SOD and MDA levels in the myocardial homogenate. The data were expressed as mean ± SEM (*n* = 10 per group). **p < 0.01, *p < 0.05 vs. Control group; ^▽▽^ *p* < 0.01, ^▽^ *p* < 0.05 vs. Control + AICAR group; ^##^ *p* < 0.01, ^#^ *p* < 0.05 vs. I/R group.

AICAR, an AMPK agonist, was first examined for its effects on MIRI. As shown in Figure 1, in the isolated mouse heart model, there were no significant differences of LVSP at baseline among the different groups. AICAR treatment significantly improved LVSP (Figure 1A), reduced the incidence of malignant arrhythmia (Figure 1C), and decreased myocardial infarct size in mice subjected to I/R treatment (Figure 1B). AICAR also reduced CK and LDH levels of the coronary effluent in the I/R group significantly (Figures 1D,E). The mRNA expression levels of *TNF-a, IL-6,* and *IL-1β* in myocardial tissue were determined, and the results showed that AICAR decreased the mRNA levels of *TNF-α, IL-1β*, and *IL-6* in myocardial tissue (Figures 1F–H). SOD is one of the most important antioxidant enzymes in myocardial tissue, and MDA reflects the level of lipid peroxidation. Our results showed that AICAR increased SOD activity (Figure 1J) and decreased MDA content after myocardial I/R (Figure 1I). Taken together, these results suggested that AICAR could alleviate I/R-induced myocardial tissue damage.

### Activation of AMPK Influenced the Expression of Mitochondrial Dynamics Related Factors in Isolated Mouse Hearts

**FIGURE 2:**
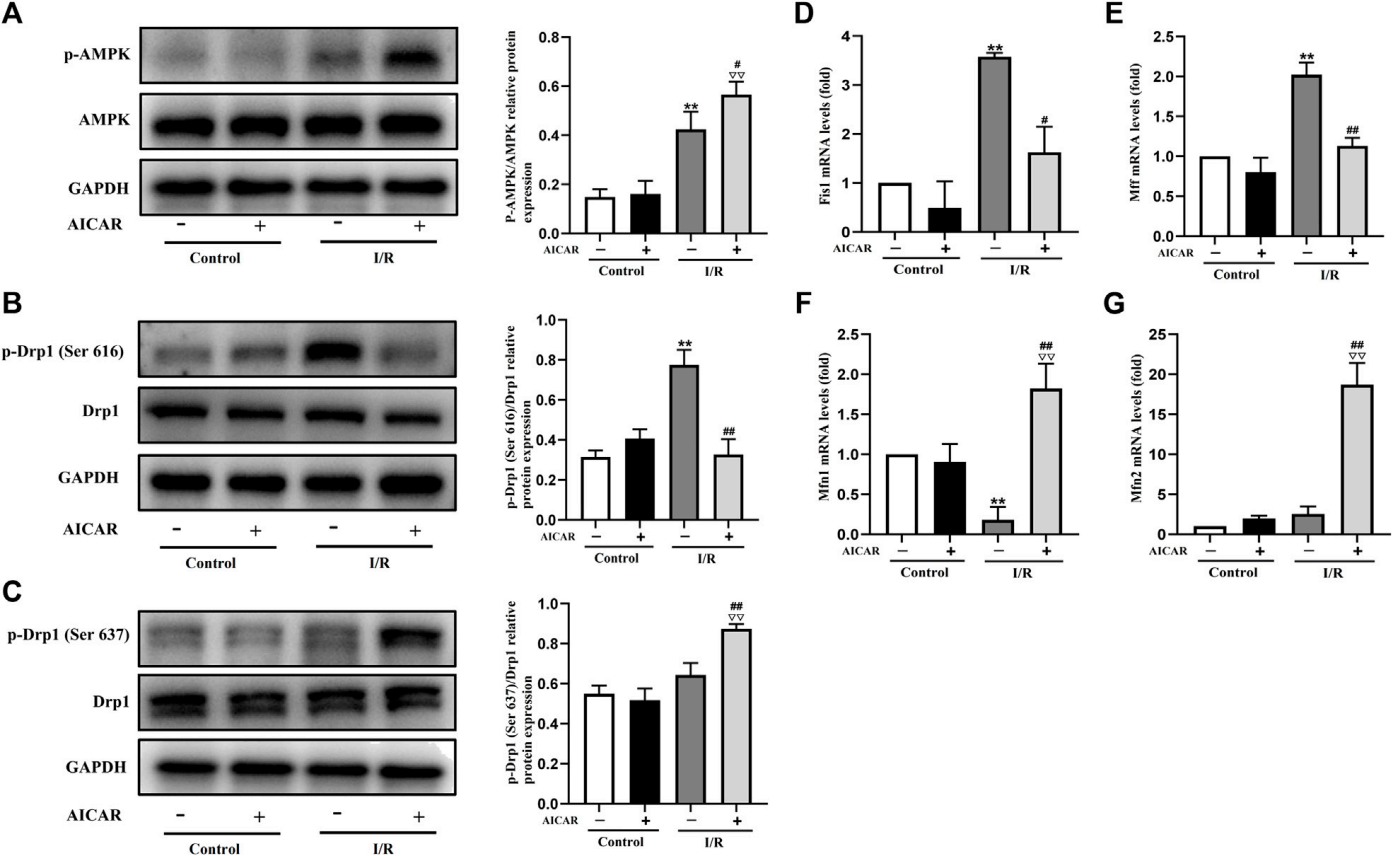
Activation of AMPK influenced the expression of mitochondrial dynamics related factors in isolated mouse hearts. **(A)** Quantitative analysis of AMPK phosphorylation in myocardium lysate. **(B,C)** Quantitative analysis of Drp1 phosphorylation in myocardium lysate. **(D–G)** The mRNA expression of mitochondrial dynamics related factors including *Fis1, Mff, Mfn1*, and *Mfn2* were detected by q-PCR. The data were expressed as mean ± SEM (*n* = 6 per group). ^**^ *p* < 0.01 vs. Control group; ^▽▽^ *p* < 0.01, ^▽^ *p* < 0.05 vs. Control + AICAR group; ^##^ *p* < 0.01, ^#^ *p* < 0.05 vs. I/R group.

Mitochondrial dynamic balance is critical for mitochondrial homeostasis. To determine the possible mechanisms of AMPK protecting against MIRI, the expression levels of mitochondrial dynamics related factors were examined by western blot or qPCR technique. As expected, AICAR significantly increased the phosphorylation level of AMPK (Figure 2A), and at the same time, the results showed that the phosphorylation level of Drp1 (Ser 637) increased (Figure 2C), while the phosphorylation level of Drp1 (Ser 616) decreased (Figure 2B). Correspondingly, we examined mitochondrial fusion and fission genes, and the results showed that AICAR could reduce the mRNA expression of mitochondrial fission factors such as Fis1 and Mff (Figures 2D,E), and promote the mRNA expression of mitochondrial fusion factors such as Mfn1 and Mfn2 (Figures 2F,G).

### Activation of AMPK Protected H9C2 Cells From H/R Induced Injury

**FIGURE 3:**
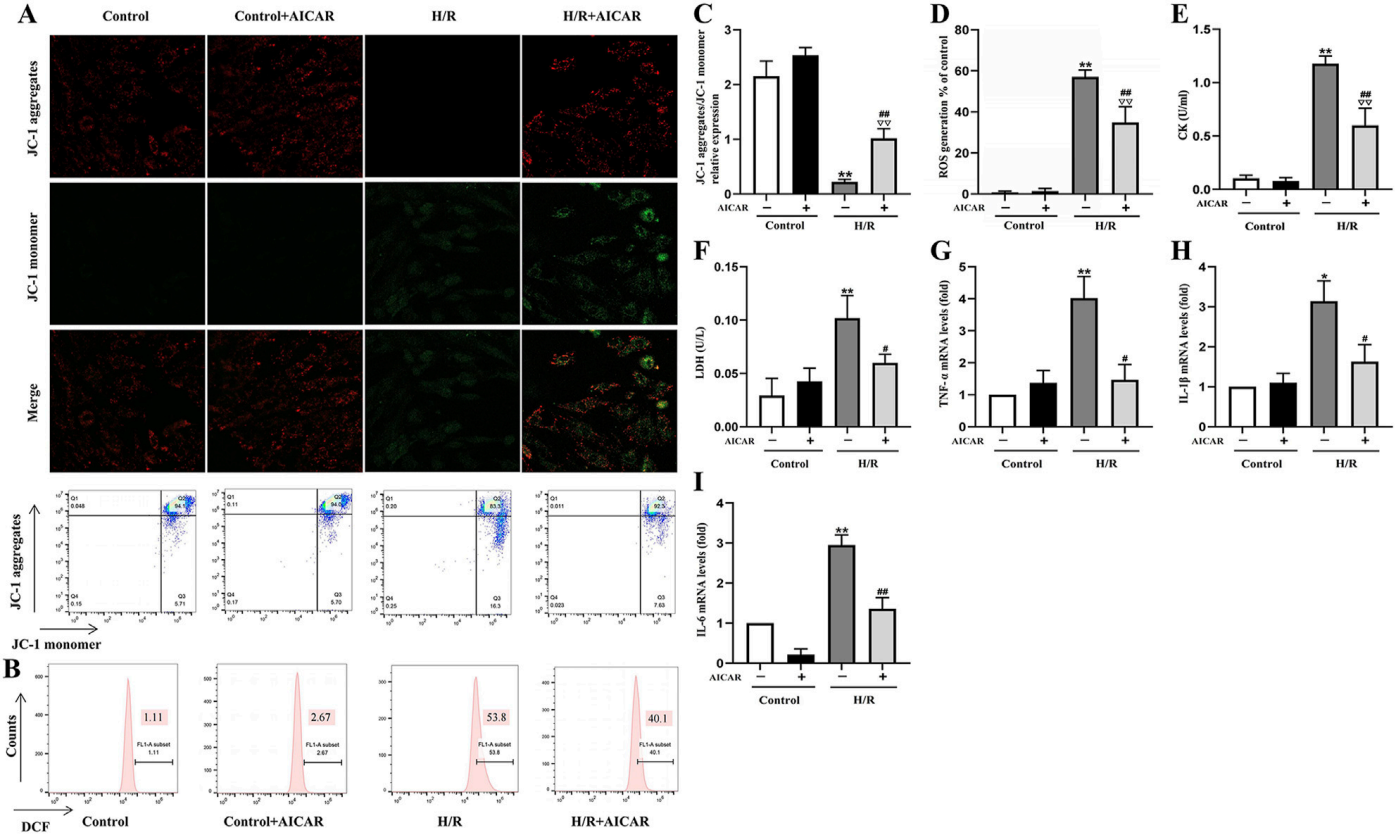
Activation of AMPK protected H9C2 cells from H/R induced injury. **(A)** Representative fluorescent and flow cytometer images of JC-1 (Green fluorescence represents monomer JC-1, and red fluorescence represents aggregate JC-1). **(B)** Representative images of mitochondrial ROS levels by flow cytometry. **(C)** Quantification of the MMP by detecting the red-to-green fluorescence intensity ratio. **(D)** AICAR decreased the ROS generation in H9C2 cells. **(E, F)** AICAR decreased the levels of CK and LDH in culture supernatant of H9C2 cells. **(G–I)** AICAR decreased the mRNA expression of *TNF-α, IL-1β*, and *IL-6* in H9C2 cells. The data were expressed as mean ± SEM (*n* = 6 per group). **p < 0.01, *p < 0.05 vs. Control group; ^∇∇^ *p* < 0.01 vs. Control + AICAR group; ^##^ *p* < 0.01, ^#^ *p* < 0.05 vs. I/R group.

As shown in Figure 3, MMP was reduced after H/R treatment in H9C2 cells (Figures 3A,C), while ROS generation was significantly increased (Figures 3B,D). AICAR treatment reversed these trends, which could improve MMP and reduce ROS levels significantly. At the same time, AICAR significantly reduced CK and LDH levels in the cell culture medium after H/R treatment (Figures 3E,F). Next, we detected the mRNA levels of inflammation factors including *TNF-α*, *IL-1β*, and *IL-6* in H9C2 cells after H/R treatment. The results showed that AICAR significantly decreased the mRNA levels of *TNF-α*, *IL-1β*, and *IL-6* in H9C2 cells (Figures 3G–I).

### Activation of AMPK Antagonised H/R Induced Mitochondrial Fission in H9C2 Cell

**FIGURE 4:**
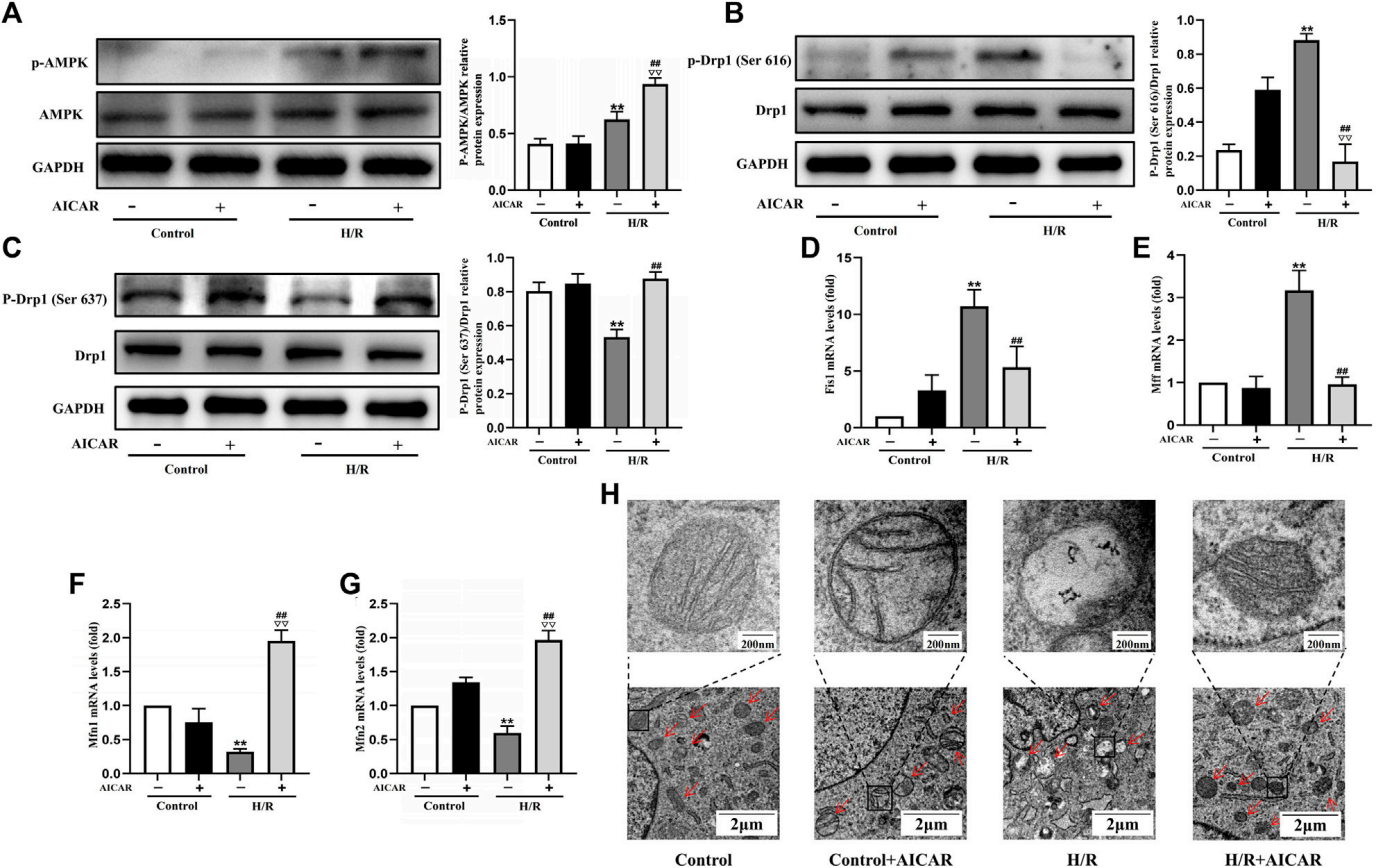
Activation of AMPK antagonised H/R induced mitochondrial fission in H9C2 cell. **(A)** Quantitative analysis of AMPK phosphorylation in cell lysate. **(B,C)** Quantitative analysis of Drp1 phosphorylation in cell lysate. **(D–G)** The mRNA expression of mitochondrial dynamics related factors including *Fis1, Mff, Mfn1*, and *Mfn2* were detected by q-PCR. **(H)** Mitochondrial morphology were measured by TEM in H9C2 cells. The data were expressed as mean ± SEM (*n* = 6 per group). ^**^ *p* < 0.01 vs. Control group; ^▽▽^ *p* < 0.01 vs. Control + AICAR group; ^##^ *p* < 0.01 vs. I/R group.

To determine the underlying mechanism of AMPK activation in protecting the cardiomyocytes, we evaluated the effect of AMPK activation on mitochondrial morphology and mitochondrial dynamic related factors in H9C2 cells. We found that H/R treatment triggered the increased phosphorylation of Drp1 at Ser 616, with a decreased phosphorylation of Drp1 at Ser 637. Compared with the H/R group, AICAR, an agonist of AMPK (Figure 4A), inhibited the phosphorylation of Drp1 at Ser 616 and increased the phosphorylation of Drp1 at Ser 637 significantly (Figures 4B,C). Next, we detected mitochondrial fusion and fission factors by qPCR, and the results were similar to those in animal experiments, which showed that AICAR reduced the expression of mitochondrial fission factors such as *Fis1, Mff* (Figures 4D,E), and promoted the expression of mitochondrial fusion factors such as *Mfn1* and *Mfn2* (Figures 4F,G). Considering that mitochondrial ultrastructure impairment is an early symbol of mitochondrial damage, mitochondrial morphology was further observed by TEM. The results showed that the outer membrane of mitochondria ruptured massively after H/R treatment, with mitochondrial fragmentation increases, and the mitochondrial cristae disappearance, even severe vacuolization. AICAR could restore the morphology of mitochondria, that is, reduced the outer membrane rupture of mitochondria and mitochondrial vacuolation, and reappearance of mitochondrial cristae (Figure 4H).

### AMPK Inhibitor Couldn’t Block the Protection of Mitochondrial Fission Inhibitor Mdivi-1 on H/R Induced Injury in H9C2 Cells

**FIGURE 5:**
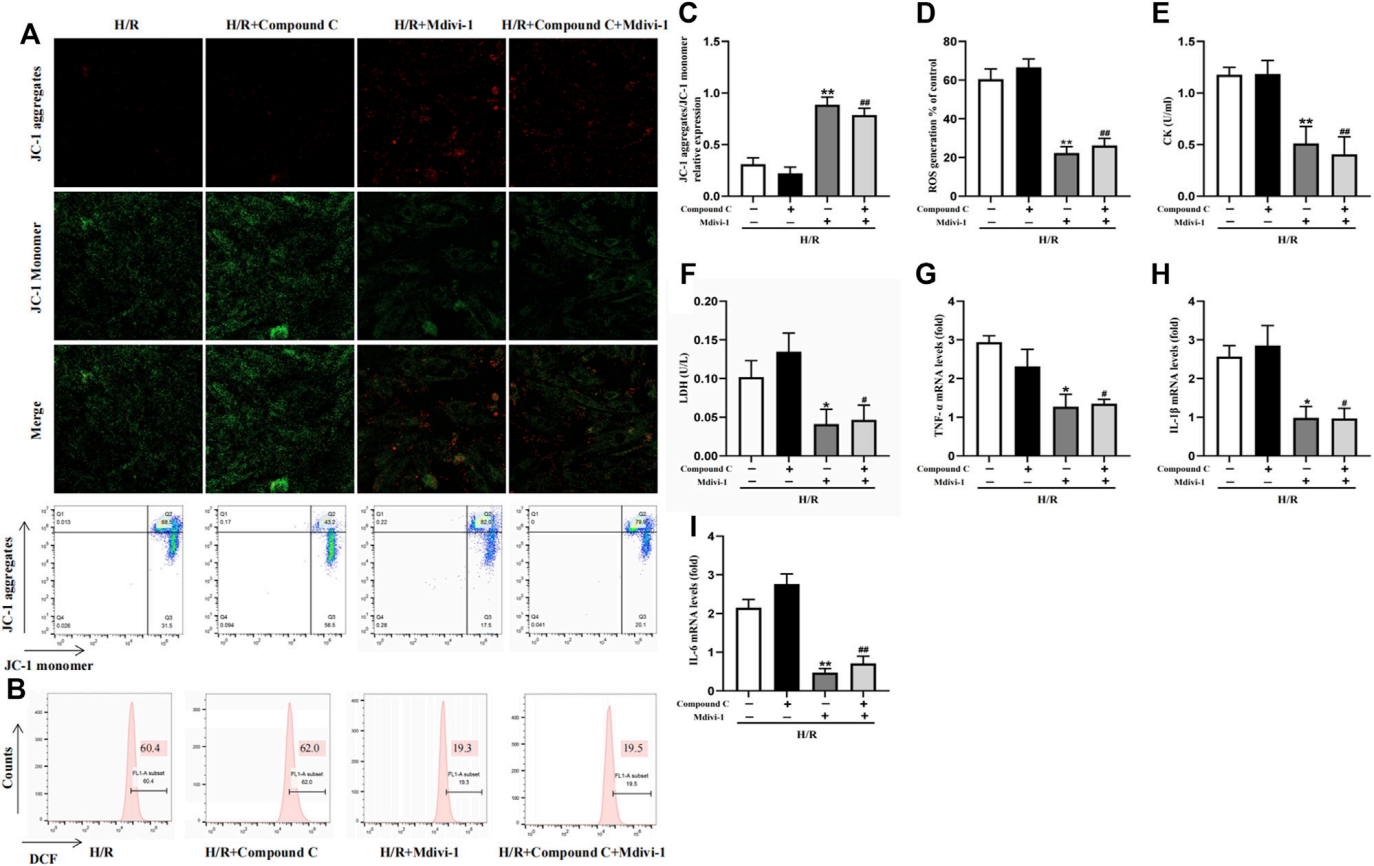
Interact effect of Compound C and Mdivi-1 on H/R induced H9C2 injury. **(A)** Representative fluorescent and flow cytometer images of JC-1 (Green fluorescence represents monomer JC-1, and red fluorescence represents aggregate JC-1). **(B)** Representative images of mitochondrial ROS levels by flow cytometry. **(C)** Quantification of the MMP by detecting the red-to-green fluorescence intensity ratio. **(D)** ROS generation in H9C2 cells. **(E,F)** The levels of CK and LDH in culture supernatant of H9C2 cells. **(G–I)** The mRNA expression of *TNF-α, IL-1β*, and *IL-6* were measured in H9C2 cells. The data were expressed as mean ± SEM (n = 6 per group). ^**^ *p* < 0.01, ^*^ *p* < 0.05 vs. H/R group; ^##^ *p* < 0.01, ^#^ *p* < 0.05 vs. H/R + Compound C group.

Mdivi-1, the selective Drp1 inhibitor, was used to verify the excessive mitochondrial fission was vital in MIRI. Compared with the H/R group, Mdivi-1 caused a sharp rise in MMP (Figures 5A,C) and a dramatic decline in ROS production (Figures 5B,D). At the same time, CK and LDH levels also decreased (Figures 5E,F). Meanwhile, Mdivi-1 treatment also decreased the mRNA levels of inflammatory factors such as *TNF-α, IL-1β*, and *IL-6* in cells (Figures 5G–I). Furthermore, after the cells were co-incubated with Mdivi-1 and Compound C, which was the AMPK inhibitor, the results showed that Compound C couldn’t block the protection effect of Mdivi-1 on H/R induced cell injury, and there was no statistically significant compared with Mdivi-1 alone.

### AMPK Inhibitor Couldn’t Block the Effect of Mdivi-1 on H/R Induced Mitochondrial Fission in H9C2 Cells

**FIGURE 6:**
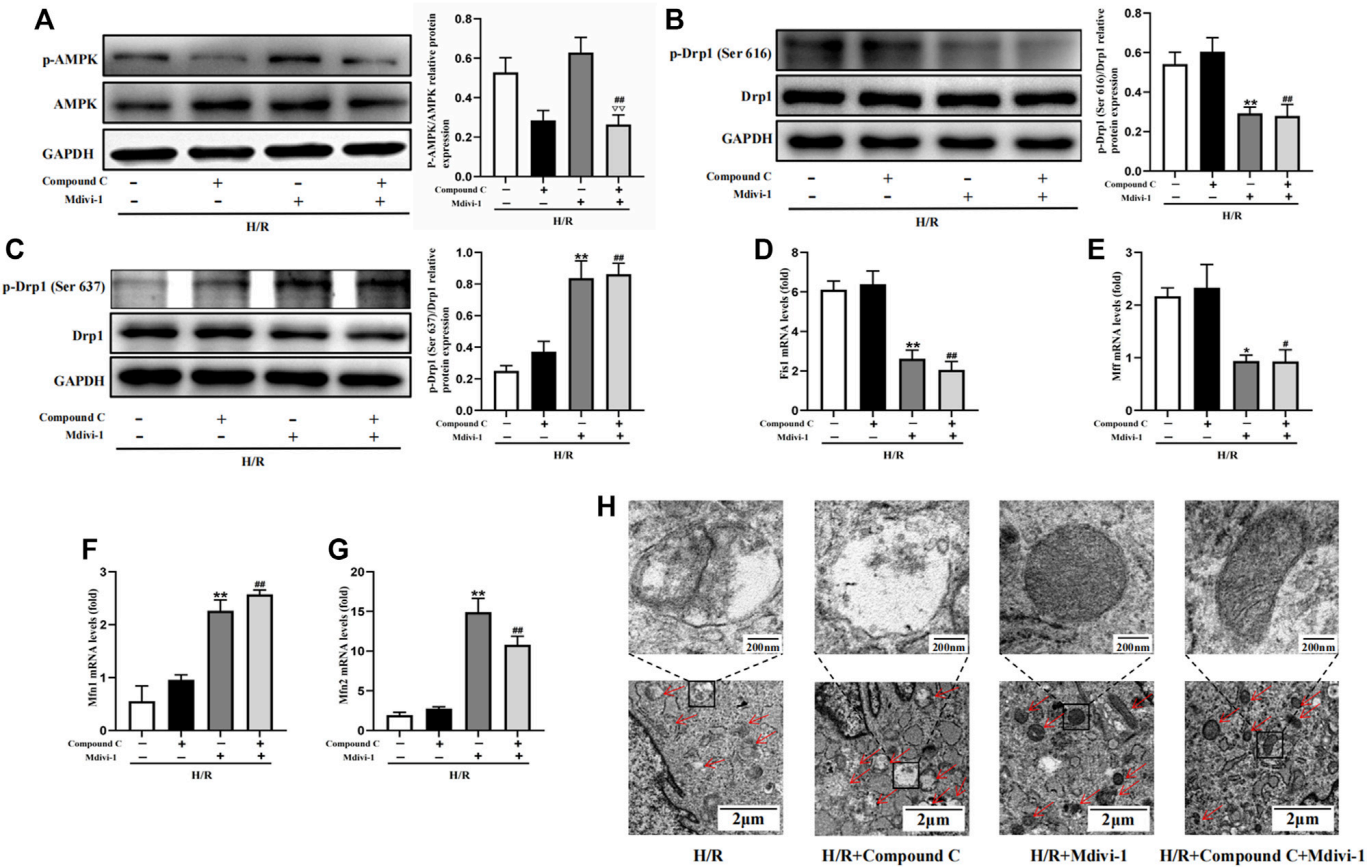
Interact effect of Compound C and Mdivi-1 on H/R induced mitochondrial fission in H9C2 cells. **(A)** Quantitative analysis of AMPK phosphorylation in cell lysate. **(B,C)** Quantitative analysis of Drp1 phosphorylation in cell lysate. **(D–G)** The mRNA expression of mitochondrial dynamics related factors including *Fis1, Mff, Mfn1*, and *Mfn2* were detected by q-PCR. **(H)** Mitochondrial morphology were measured by TEM in H9C2 cells. The data were expressed as mean ± SEM (*n* = 6 per group). ^**^ *p* < 0.01, ^*^ *p* < 0.05 vs. H/R group; ^##^ *p* < 0.01, ^#^ *p* < 0.05 vs. H/R + Compound C group. ^▽▽^ *p* < 0.01 vs. I/R + Mdivi-1 group.

Firstly, Mdivi-1 was used to investigate its effects on the expression of mitochondrial dynamic related factors. Compared with the H/R group, Mdivi-1 treatment reduced the phosphorylation of Drp1(Ser 616) significantly, and increased the phosphorylation of Drp1(Ser 637) (Figure 6B,C). Moreover, the qPCR results showed that Mdivi-1 reduced the mRNA expression of mitochondrial fission factors such as *Fis1* and *Mff* (Figure 6D, E), and promoted the mRNA expression of mitochondrial fusion factors such as *Mfn1* and *Mfn2* (Figure 6F,G). Secondly, as the selective AMPK inhibitor, Compound C was used to further verify that Drp1-mediated mitochondrial dynamics was the downstream of AMPK signaling. As expected, Compound C inhibited the phosphorylation of AMPK in cells (Figure 6A), while it couldn’t reverse the effects of Mdivi-1 on Drp1 signaling and mitochondrial dynamic related factors when co-incubated with Mdivi-1 and Compound C in H9C2 cells.

Furthermore, mitochondrial morphology was further observed by TEM, the results showed that Mdivi-1 could restore the mitochondrial morphology after H/R treatment, such as reducing the outer membrane rupture and vacuolation of mitochondria, and reappearance of mitochondrial cristae. When co-incubation with Mdivi-1 and Compound C, the latter couldn’t reverse the protective effects of Mdivi-1 on mitochondrial morphology (Figure 6H).

## Discussion

AMPK, a key energy sensor that regulates cellular metabolism to maintain energy homeostasis, has been reported to have multiple organ protection functions (Trefts and Shaw, 2021). Although previous studies have indicated that the AMPK agonist AICAR decreased cardiomyocyte apoptosis, improved cardiac function, and inhibited MIRI (Fan et al., 2021), the specific mechanism remains unclear. In the present study, the effects of AICAR were examined to determine whether the activation of AMPK signaling could protect the heart against MIRI, and to explore the potential mechanism. The results showed that AICAR could protect heart or H9C2 cells from I/R or H/R-induced injury by activating the AMPK signaling, which exerted a beneficial effect on mitochondrial dynamics, enhanced mitochondrial fusion, and inhibited mitochondrial fission, which ultimately reduced the myocardial infarct size and improved left ventricular dysfunction.

Unbalanced mitochondrial dynamics is a fatal factor causing mitochondrial dysfunction and cardiomyocyte apoptosis during MIRI (Maneechote et al., 2019; Xue et al., 2020). Drp1 is the main functional protein mediating mitochondrial dynamic changes. Under stress, Drp1 can be recruited to the outer membrane of mitochondria and thus trigger the division of mitochondria (Zheng et al., 2019; Sun et al., 2021). The function of Drp1 is regulated by phosphorylation, acetylation, glycosylation, etc. (Hu et al., 2020; Sun et al., 2021). Studies have shown that phosphorylation of Drp1 at Ser 616 accelerated its recruitment to the mitochondrial membrane, while phosphorylation at Ser 637 hindered this process (Wu et al., 2020). Excessive mitochondrial translocation of Drp1 not only triggered mitochondrial fragmentation elevation, but also led to increased mitochondrial ROS generation. Meanwhile, Cytochrome C (Cytc) was released into the cytoplasm, therefore triggering inflammatory response and cell apoptosis (Xu et al., 2016; Sun et al., 2021). Given the detrimental effect of sustained mitochondrial fission in ischemic heart disease, exploring new ways to attenuate Drp1-mediated mitochondrial fission is of clinical interest to combat MIRI.

In the present study, we confirmed that I/R or H/R treatment led to an imbalance of mitochondrial dynamic genes. As the qPCR results showed, the expression of mitochondrial fission factors such as *Mff* and *Fis1* increased significantly, conversely, the expression of mitochondrial fusion factors such as *Mfn1* or *Mfn2* decreased. Meanwhile, mitochondrial morphology shown by TEM further confirmed that mitochondrial fragmentation increased and mitochondria were damaged. This meant that I/R or H/R treatment induced imbalance of mitochondrial dynamic and function. The AMPK pathway was the classic downstream target of AICAR (Ahmad et al., 2021). We found AICAR could reverse these imbalances, which inhibited the expression of mitochondrial fission factors such as *Mff* and *Fis1*, and enhanced the expression of mitochondrial fusion factors such as *Mfn1* and *Mfn2*, and then maintaining the balance of mitochondrial dynamic and function, thereby inhibiting mitochondrial ROS production and inflammatory reactions. At the protein level, AICAR inhibited the phosphorylation of mitochondrial fission-associated proteins Drp1 (Ser 616), and enhanced the phosphorylation of mitochondrial fusion-associated proteins Drp1 (Ser 637) both in the mouse model of MIRI and in the cell model of H/R. Overall, these results indicated the association between AMPK signaling and Drp1 mediated mitochondrial dynamics, which was consistent with the previous reports (Sun et al., 2021).

To further verify that Drp1-mediated mitochondrial dynamics was regulated by AMPK signaling, firstly, we used Mdivi-1 to pretreat the cells, which was the mitochondrial fission inhibitor. Results showed that Mdivi-1 protected cardiomyocytes and restored the mitochondrial dynamics balance just as the AICAR did. Secondly, we used Compound C, a selective inhibitor of AMPK, we found that the protective effects of Mdivi-1 on cardiomyocytes and mitochondria were almost unaffected when they were co-incubated. These studies indicated that it was possible to regulate the phosphorylation of Drp1 as a potential therapeutic target for protecting the mitochondria and cardiomyocytes under MIRI, and AICAR suppressed Drp1-mediated mitochondrial damage dependent on AMPK signaling.

## Conclusion

In summary, in the present report, the experiments were performed to investigate the protective mechanism of AMPK activation on MIRI. Our findings demonstrated that AICAR protected cardiomyocytes and mitochondria by activating AMPK signaling, which depended on Drp1-mediated mitochondrial dynamics. Our results not only provide a theoretical foundation for exploring the mechanism but also put forward a promising treatment target for MIRI.

## Data Availability

The original contributions presented in the study are included in the article/Supplementary Material, further inquiries can be directed to the corresponding author.
