# Corrigendum: AMPK activation alleviates myocardial ischemia-reperfusion injury by regulating Drp1-mediated mitochondrial dynamics

**DOI:** 10.3389/fphar.2024.1502512

**Published:** 2024-10-11

**Authors:** Jingxia Du, Hongchao Li, Jingjing Song, Tingting Wang, Yibo Dong, An Zhan, Yan Li, Gaofeng Liang

**Affiliations:** ^1^ Pharmacy Department, School of Basic Medical Sciences, Henan University of Science and Technology, Luoyang, China; ^2^ Pathology Department, School of Basic Medical Sciences, Henan University of Science and Technology, Luoyang, China

**Keywords:** AMPK, DRP1, mitochondrial dynamics, myocardial ischemia/reperfusion injury, ROS, inflammatory factors

In the published article, there was an error in Figure 3 as published. The fluorescent image for the H/R group in Figure 3A was mis-assigned. The corrected Figure 3 and its caption appear below.

**FIGURE 3:**
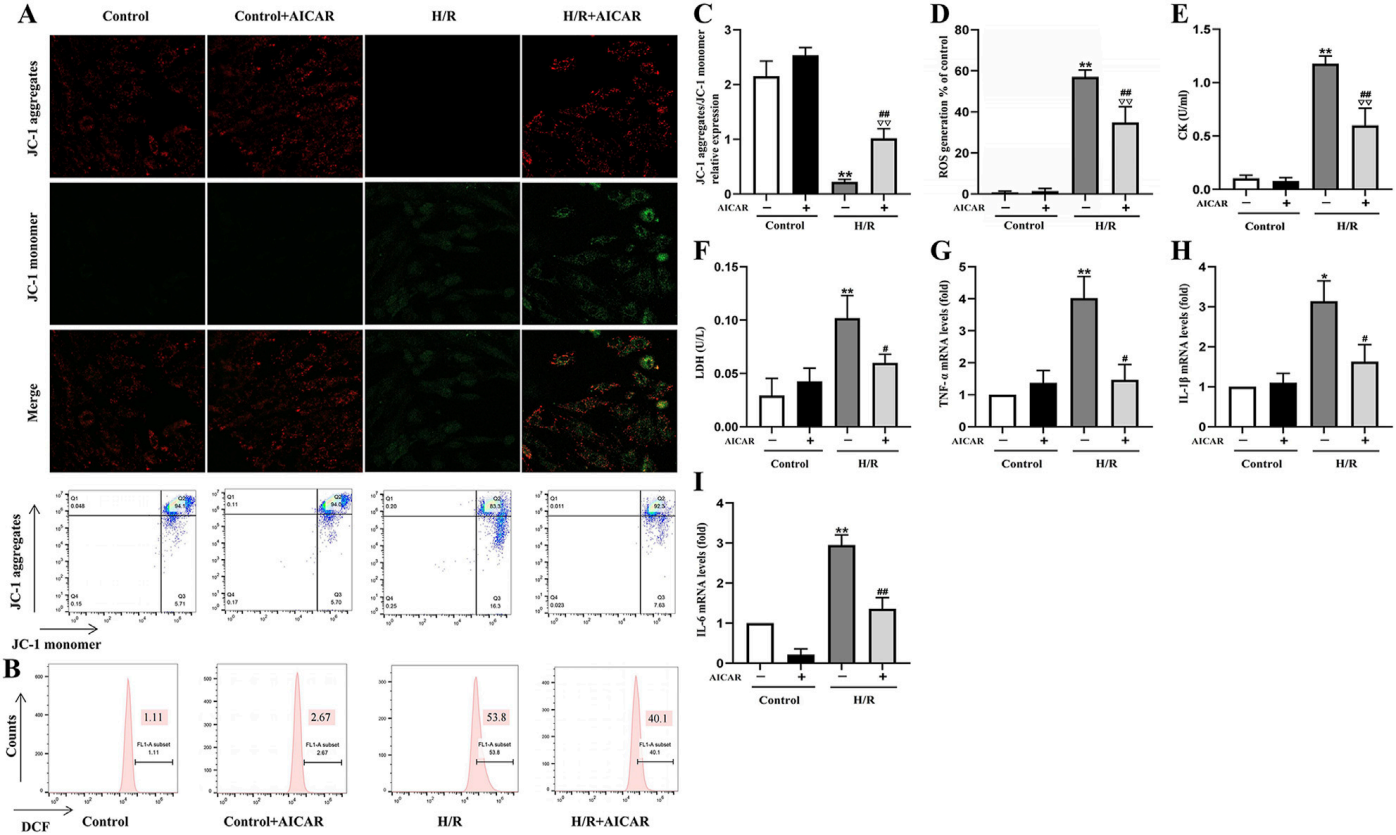
Activation of AMPK protected H9C2 cells from H/R induced injury. **(A)** Representative fluorescent and flow cytometer images of JC-1 (Green fluorescence represents monomer JC-1, and red fluorescence represents aggregate JC-1). **(B)** Representative images of mitochondrial ROS levels by flow cytometry. **(C)** Quantification of the MMP by detecting the red-to-green fluorescence intensity ratio. **(D)** AICAR decreased the ROS generation in H9C2 cells. **(E, F)** AICAR decreased the levels of CK and LDH in culture supernatant of H9C2 cells. **(G–I)** AICAR decreased the mRNA expression of *TNF-α, IL-1β*, and *IL-6* in H9C2 cells. The data were expressed as mean ± SEM (*n* = 6 per group). ^**^ *p* < 0.01, ^*^ *p* < 0.05 vs. Control group; ^▽▽^ *p* < 0.01 vs. Control + AICAR group; ^##^ *p* < 0.01, ^#^ *p* < 0.05 vs. I/R group.

The authors apologize for this error and state that this does not change the scientific conclusions of the article in any way. The original article has been updated.

