# Supplementary figures and images for: AMPK Activation Alleviates Myocardial Ischemia-Reperfusion Injury by Regulating Drp1-Mediated Mitochondrial Dynamics
Source: Front Pharmacol. 2022 Jul 4;13:862204. doi: 10.3389/fphar.2022.862204 (PMC9289369; doi:10.3389/fphar.2022.862204)

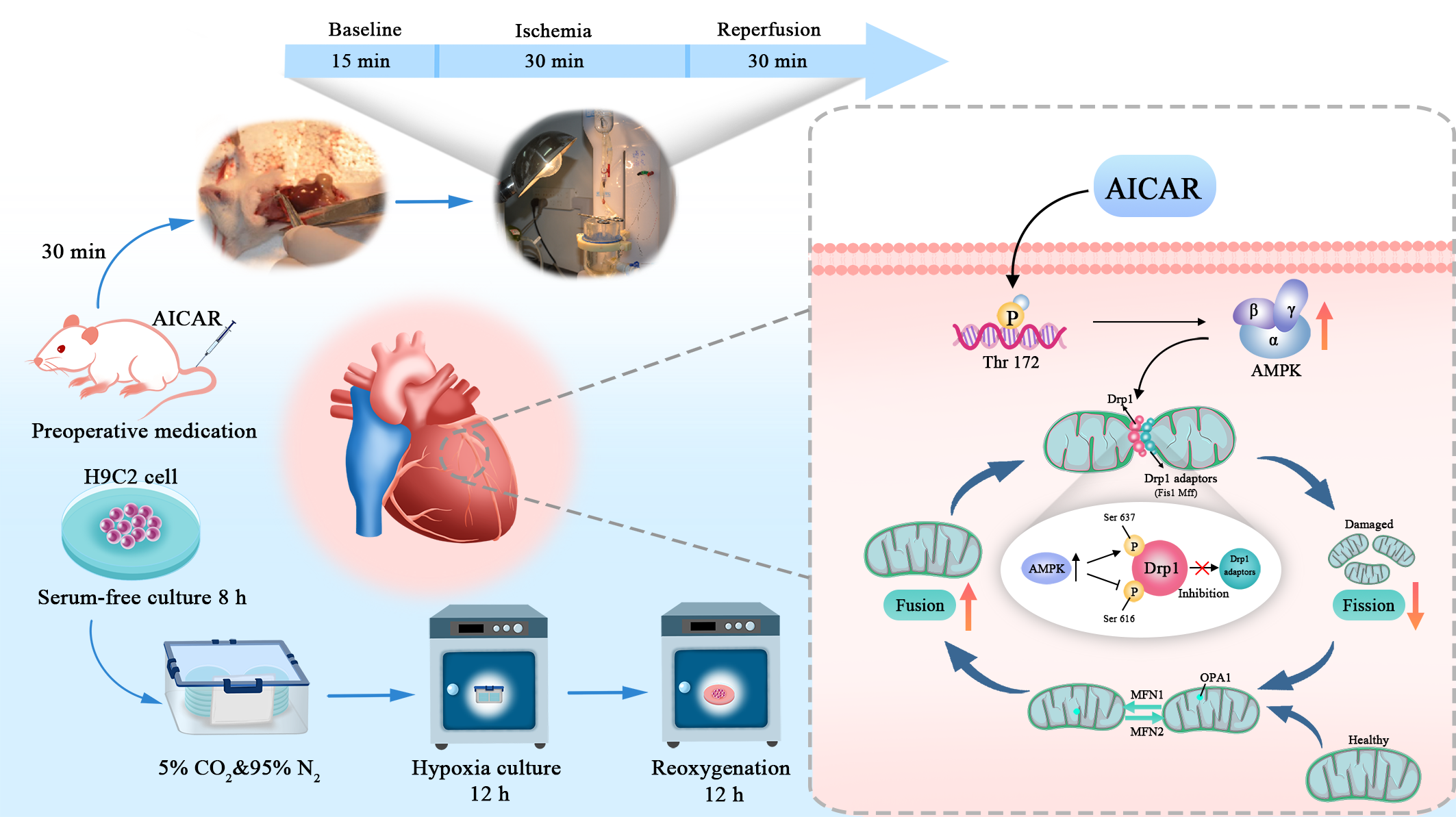

Supplement: Supplementary file 1 [file Image1.TIF]
