# Supplementary material for: AMPK Activation Alleviates Myocardial Ischemia-Reperfusion Injury by Regulating Drp1-Mediated Mitochondrial Dynamics
Source: Front Pharmacol. 2022 Jul 4;13:862204. doi: 10.3389/fphar.2022.862204 (PMC9289369; doi:10.3389/fphar.2022.862204)
Supplement: Supplementary file 2 [file DataSheet1.docx]

**Primer sequences of rat used in qPCR analysis**

| **Gene** | **Sequence** |
| --- | --- |
| Mfn1 | Forward: 5'- CGTGGCAGCAGCAGAGAAGAG-3' |
|  | Reverse: 5'-CTAGTTCCTCCTCCAGTGCCTCCTCC-3' |
| Mfn2 | Forward: 5'- TCCACAGCCATTGCCAGTTCAC-3' |
|  | Reverse: 5'-GGAAGAAGGACACAGACACGCC-3' |
| Fis1 | Forward: 5'-CCAGAACAACCAGGCCAAGGAG-3' |
|  | Reverse: 5'- GACCTGAGTAACCTGACCGACAC-3' |
| Mff | Forward: 5′-ACATGCGCATTGGAGCAGTA-3′ |
|  | Reverse: 5′-GCCCCACTCACCAAATGAGA-3′ |
| TNFɑ | Forward: 5′-TCTCAAAACTCGAGTGACAAGC-3′ |
|  | Reverse:5′-GGTTGTCTTTGAGATCCATGC-3′ |
| IL-1β | Forward: 5′-TACCTATGTCTTGCCCGTGGA-3′ |
|  | Reverse:5′-ATCATCCCACGAGTCACAGAGG-3′ |
| IL-6 | Forward: 5′-ACTTCACAAGTCGGAGGCTT-3′ |
|  | Reverse: 5′-AGTGCATCATCGCTGTTCAT-3′ |
| Actin | Forward: 5′-TGTCACCAACTGGGACGATA-3′ |
|  | Reverse: 5′-AAACTCTGGAAGTTGTGGGG-3′ |

**Primer sequences of mouse used in qPCR analysis**

| **Gene** | **Sequence** |
| --- | --- |
| Mfn1 | Forward: 5'-GAACCACCAAGGAGTGTGGAA-3' |
|  | Reverse: 5'-CCCTCCCATGAAAAGGAAACAT-3' |
| Mfn2 | Forward: 5'-AAAAACGCAATGTCCCTGCT-3' |
|  | Reverse: 5'-CTTCTGTGGTAACGGGGTCC-3' |
| Fis1 | Forward: 5'- TGTCCAAGAGCACGCAGTTT -3' |
|  | Reverse: 5'- GAGCAGCACGATGCCTTTAC -3' |
| Mff | Forward: 5'-CGTGCTCTCAGCCAACCA -3' |
|  | Reverse: 5'- TGCCAACTGCTCGGATTTCT-3' |
| TNFɑ | Forward: 5′-CATCTTCTCAAAATTCGAGTGACAA-3′ |
|  | Reverse:5′-TGGGAGTAGACAAGGTACAACCC-3′ |
| IL-1β | Forward: 5′-CCGTGGACCTTCCAGGATGA-3′ |
|  | Reverse:5′-GGGAACGTCACACACCAGCA-3′ |
| IL-6 | Forward: 5′-AGTTGCCTTCTTGGGACTGA-3′ |
|  | Reverse: 5′-TCCACGATTTCCCAGAGAAC-3′ |
| Actin | Forward: 5′-GTGACGTTGACATCCGTAAAGA-3′ |
|  | Reverse: 5′-GCCGGACTCATCGTACTCC-3′ |
